# Efficacy and Tolerability of Methotrexate and Methylprednisolone in a Comparative Assessment of the Primary and Long-Term Outcomes in Patients with Pulmonary Sarcoidosis

**DOI:** 10.3390/diagnostics11071289

**Published:** 2021-07-19

**Authors:** Volodymyr Gavrysyuk, Ievgenia Merenkova, Yaroslav Dziublyk, Nataliia Morska, Nataliia Pendalchuk, Olesia Bychenko, Nataliia Vlasova

**Affiliations:** 1 Department of Interstitial Lung Diseases, National Institute of Phthisiology and Pulmonology Named after F. G. Yanovsky, 10, M. Amosova str., 03038 Kyiv, Ukraine; gavrysyuk@ukr.net (V.G.); dziublyk@gmail.com (Y.D.); nmorska@gmail.com (N.M.); pendalchuk@gmail.com (N.P.); olesia_bychenko@ukr.net (O.B.); 2 Department of Differential Diagnosis of Tuberculosis and Nonspecific Lung Deseases, National Institute of Phthisiology and Pulmonology Named after F. G. Yanovsky, 10, M. Amosova str., 03038 Kyiv, Ukraine

**Keywords:** pulmonary sarcoidosis, methotrexate, methylprednisolone, outcomes

## Abstract

Background: There is insufficient information in the literature on the comparative efficacy and tolerability of methotrexate (MTX) and methylprednisolone (MP) in patients with pulmonary sarcoidosis in assessing primary outcomes and the relapse rate. Purpose: The aim of our study was to evaluate primary and long-term outcomes of using MTX and MP in patients with pulmonary sarcoidosis. Methods: A total of 143 patients with newly diagnosed pulmonary sarcoidosis, verified by high-resolution computed tomography (CT) data, were examined. Corticosteroid (CS) therapy was used in 97 patients using MP at a dose of 0.4 mg/kg body weight for 4 weeks, followed by a dose reduction to 0.1 mg/kg by the end of the sixth month. The total duration of CS therapy was 12 months on average. Forty-six patients were treated with MTX at a dose of 10 mg/week (28) and 15 mg/week (18) per os for 6 to 12 months. The study of the relapse rate was conducted within 12 months after the CT data normalization in 60 patients after CS therapy and in 24 after MTX treatment. Results: MP treatment was successfully completed in 68 (70.1%), and MTX in 29 (60.4%) patients. In five MP patients (5.2%) and in five (10.9%) MTX, treatment was discontinued due to serious side effects. In seven (7.2%) MP patients and ten (21.7%) MTX patients, treatment required additional therapy due to the lack of efficacy. Progression with MP treatment (17–17.5%) was more common than with MTX (2–4.3%; Chi square = 4.703, *p* = 0.031). Relapses after MP therapy were observed in 26 (43.3%) patients, and after MTX therapy in 2 (8.3%; Chi square = 9.450, *p* = 0.003). Conclusion: In patients with pulmonary sarcoidosis, MTX monotherapy does not differ significantly from MP monotherapy in terms of the level of efficacy and the rate of serious side effects. Increasing the MTX dose from 10 to 15 mg/week accelerates the rate of regression of sarcoidosis, improves treatment efficacy, and does not affect the rate of serious side effects. When using MTX, there is a significant decrease in the incidence of treatment resistance and the relapse rate.

## 1. Introduction

Sarcoidosis is a multisystemic disease of unknown nature, characterized by the formation of noncaseating epithelioid cell granulomas in the affected organs [1,2,3].

Usually, sarcoidosis affects young and middle-aged people. In more than 90% of patients, it is manifested as intrathoracic lymphadenopathy and lung parenchymal lesions [4]. The eyes, skin, liver, spleen, other groups of lymph nodes, salivary glands, heart, nervous system, muscles, bones, and other organs may also be involved [5,6]. The lungs and intrathoracic lymph nodes are affected in more than 90% of patients [4,7].

If there are indications for systemic pharmacotherapy (dyspnea, cough, pulmonary function impairment, involvement of the heart, nervous system, eyes, hypercalcemia, as well as all cases of disease progression [1,8,9]), first-line agent corticosteroids (CS) are prescribed [1,8,10,11]. Their efficacy in assessing the immediate results has been proven in randomized trials [12,13]. At the same time, the study of the long-term outcomes of CS therapy showed a high relapse rate, which is currently one of the most acute problems in the management of patients with pulmonary sarcoidosis [14]. The relapse rate of sarcoidosis ranges from 13% to 75% depending on the study population [15,16].

In three categories of patients, effective and safe CS therapy is not possible. The first of them includes patients with contraindications to CS treatment, the second one includes patients with serious side effects of CS requiring discontinuation of the drug, and the third one includes patients with resistance to CS therapy [17].

In cases of contraindications, serious side effects, and resistance to CS therapy, second-line agents are prescribed—such immunosuppressants as methotrexate, azathioprine and leflunomide [18,19,20,21]. Currently, methotrexate (MTX) is most widely used in clinical practice as a drug with an acceptable safety profile and efficacy level in assessing primary outcomes [18]. However, encouraging data on the low relapse rate of pulmonary sarcoidosis after MTX therapy have only been published in recent years [22,23].

The aim of this work was to study the efficacy and tolerability of methotrexate and methylprednisolone as monotherapy in a comparative assessment of short-term and long-term outcomes in patients with newly diagnosed pulmonary sarcoidosis.

## 2. Materials and Methods

### 2.1. Study Groups

The study included 143 patients with pulmonary sarcoidosis with clinical symptoms and/or impaired ventilation function and lung diffusion capacity—60 males (41.9%) and 83 females (58.1%), with a mean age of 42 (range, 22–57) years. The diagnosis of pulmonary sarcoidosis was verified by high resolution computed tomography (CT) using the criteria described by Veltkamp M., Grutters J. C. [4]; a surgical lung biopsy was performed in 7 cases due to the presence of atypical CT signs. All patients had bilateral hilar lymphadenopathy and/or mediastinal lymphadenopathy in combination with lung parenchymal lesions (X-ray stage II) [1].

When selecting patients, two principles were strictly adhered to: firstly, the study included patients with only newly diagnosed sarcoidosis without any prior specific therapy; secondly, an important selection criterion which allowed with the maximum possibility to exclude patients with a long-term ongoing disease was the absence of CT signs of interstitial pulmonary fibrosis.

The ventilation function of the lungs was assessed by spirometry and bodyplethysmography, and the diffusing capacity of the lung for carbon monoxide (DLCO) by the single-breath method.

General weakness and increased fatigue were the most frequent subjective manifestations of sarcoidosis at the onset of the disease.

The second most frequent were respiratory symptoms—coughs, mostly dry, and dyspnea. Chest pains were relatively rare (4.2% of cases). A temperature rise predominantly to subfebrile values was observed in almost every sixth patient (16.1%). During auscultation, pathological findings in the lungs were not detected, as a rule.

Extrapulmonary manifestations of sarcoidosis were observed in 31.5% of patients. In this case, arthralgia, hepatomegaly, lesions of the skin and peripheral lymph nodes were most often noted.

Moderate impairments of the pulmonary ventilation function were observed in 29 patients (20.3%). Among them, 16 patients (11.2%) had a predominantly obstructive type of ventilation disorder, and 13 patients (9.1%) had a predominantly restrictive type. As a rule, there was a slight decrease in DLCO in patients with impaired pulmonary ventilation.

### 2.2. Study Design

CS therapy was carried out in 97 patients. MP was used at a dose of 0.4 mg/kg of body weight, which corresponds to the maximum dose in the category of medium doses (30 mg of prednisolone in equivalent for a patient weighing 60 kg) [24] within 4 weeks. The dose was then tapered within 8 weeks so that by the end of the third month, it was 0.2 mg/kg. After 3 months from the start of treatment, its efficacy was assessed. With positive dynamics of clinical and CT data, the dose was gradually tapered to 0.1 mg/kg by the end of the 6th month. During the subsequent period of treatment, the dose remained unchanged. The total duration of CS therapy was at least one year.

MTX was used as an initial treatment in 46 patients. A total of 28 patients had relative contraindications to long-term CS therapy: diabetes mellitus—5 patients, arterial hypertension—9 patients, osteochondrosis—4 patients, obesity (body mass index > 30 kg/m^2^)—6 patients, peptic ulcer—2 patients, and mental disorders—2 patients. Eighteen patients were included in the MTX arm due to a lack of compliance with long-term CS therapy, that is, alternative treatment was the patient’s personal preference.

In accordance with the recommendations of the experts of the World Association of Sarcoidosis and Other Granulomatous Disorders (WASOG) [18], MTX for sarcoidosis is prescribed at doses of 5 to 15 mg per week. We used the drug at two doses—10 mg/week (28 patients) and 15 mg/week (18 patients) per os—to study the possible dependence of the drug efficacy and tolerability on the dose. The treatment duration was at least 6 months.

The outcomes of using MP and MTX were assessed as:

- successful completion of treatment—the disappearance of clinical symptoms with the normalization of PFTs and CT data;
- lack of efficacy—the absence of positive dynamics of CT symptoms (stabilization) during the last 3 months of treatment, despite the fact that there may be a good clinical, functional, and radiographic response to the initial therapy;
- progression during treatment (resistance to therapy)—worsening of symptoms at any stage of treatment;
- serious side effects—side effects which require the drug discontinuation.

Possible side effects of MP were monitored taking into account clinical symptoms, blood pressure dynamics and blood glucose levels. In the group of patients taking MTX, a blood test was performed to determine the content of leukocytes, platelets, alanine aminotransferase (ALT), and creatinine in the blood 2 weeks after the start of therapy, and then once a month during the treatment period.

The relapse rate was studied 6 and 12 months after the successful completion of treatment assessing possible clinical symptoms and CT data.

### 2.3. Statistics

All results for the categorical variables are presented as a number and a percentage. A Chi-squared test was used to compare data of categorical variables and Fisher’s exact test was used as appropriate. The null hypothesis for the Chi-squared test is that no relationship exists on the categorical variables; they are independent. The hypothesis about the correspondence of the studied numerical samples to the normal distribution law was tested using the D’Agostino’s D criterion. Normally distributed continuous variables are expressed as a mean with standard deviation. Comparisons were made between groups using the Student’s *t*-test. All tests were two-sided with the significance level set to *p* < 0.05.

## 3. Results

### 3.1. Baseline Characteristics

Assessment primary outcomes, used for evaluation of the efficacy and safety of MP and MTX, were based on the change of clinical, functional, and CT symptoms. During the initial stage of the study, we conducted a comparative analysis of the baseline clinical symptoms of the patients (Table 1).

In the group of patients who received corticosteroid therapy (*n* = 97), there were 54 (55.7%) females and 43 (43.3%) males; the mean age was 40 years (range, 22–50 years). In the MTX treatment group (*n* = 46), women were also more prevalent (29–63.0%); the mean age was 46 years (range, 28–57 years).

Comparative analysis of baseline demographical data, clinical, functional and radiological symptoms demonstrated no significant differences between study groups.

### 3.2. Efficacy and Tolerability of MP and MTX in a Comparative Assessment of Primary Outcomes

Comparative efficacy analysis showed that immunosuppressive therapy with MTX does not significantly differ from CS therapy with MP in terms of the rate of successful completion of treatment (Table 2).

An overall Chi-squared test was also performed using 4 by 2 tables: the results indicate a relationship between primary outcomes and drug choice: the chi-square statistic is 11.3005, the *p* value is 0.010, and the result is significant at *p* ˂ 0.05; statistical power is 86.7%.

At the same time, the average duration of CS therapy until clinical remission with normalization of CT data (12.7 ± 3.0 months) was higher than that in patients taking MTX (10.8 ± 2.7); *p* ˂ 0.05; statistical power—96.6%.

Resistance to CS therapy (progression during treatment) was observed in 17 (17.5%) patients, which was significantly more frequent than in patients taking MTX (2–4.3%; Chi-square = 4.703, *p* = 0.031). At the same time, in two patients, progression began already at the initial stage of treatment with the use of MP at a dose of 0.4 mg/kg of body weight. Two cases of progression were observed during treatment with MTX at a dose of 10 mg/week.

Lack of efficacy was observed more often in patients after MTX therapy in almost all cases when using the drug at a dose of 10 mg/week.

Serious side effects of MP requiring drug discontinuation occurred in five patients—osteoporosis with clinical manifestations in three patients and steroid diabetes in two patients—all cases after 6 months of therapy. At the first stage of CS therapy, when relatively high doses of MP were used, side effects such as weight gain, a tendency to peripheral oedema, mild hyperglycemia, hyperexcitability, and sleep disturbances were often observed, which disappeared or decreased with subsequent therapy in an MP dose reduction mode.

In the group of patients taking MTX, serious side effects with subsequent drug discontinuation were reported in five patients: methotrexate-induced pneumonitis at the 11th month of MTX therapy at a dose of 10 mg/week in one case; in two patients—an increase in ALT more than three times compared to the normal level; and in two patients—leukocytopenia with a leukocyte level below 3.5 × 10^9^/L after long-term treatment (more than 6 months). It should be noted that gastrointestinal disorders in the treatment of MTX per os were successfully stopped by dividing the therapeutic dose into two doses within a 12 h period [18], by adding folic acid (5 mg/week), or by changing the route of administration to parenteral.

Thus, in patients with newly diagnosed pulmonary sarcoidosis, MTX monotherapy does not significantly differ from MP monotherapy in terms of successful completion of treatment with normalization of CT data and the frequency of side effects. However, cases of insufficient efficacy (stabilization) after MTX therapy are observed more often.

At the same time, when using MTX, a significant decrease in the frequency of treatment resistance is observed.

### 3.3. Efficacy and Tolerability of MTX on Depending on the Dose

Table 3 shows the results of a comparative assessment of primary outcomes in 28 patients with pulmonary sarcoidosis after MTX therapy at a dose of 10 mg/week, and in 18 patients receiving MTX at a dose of 15 mg/week.

An improvement in the indicators of the treatment efficacy was noted with the use of MTX at a dose of 15 mg/week. At the same time, no significant dependence of the rate of treatment resistance and the number of serious side effects on the MTX dose has been established.

We did not perform the overall Chi-squared test using 4 by 2 tables to investigate the relationship between outcomes and different MTX dosages due to the inherent limitations of this method associated with an insufficient number of observations.

In patients treated with MTX at a dose of 15 mg/week, the average duration of therapy until clinical remission with normalization of CT data (10.1 ± 2.1 months) was less than in patients receiving MTX at a dose of 10 mg/week (12.8 ± 4.2; *p* ˂ 0.05); statistical power—82.3%. That is, increasing the MTX dose to 15 mg/week accelerates the rate of regression of sarcoidosis.

As an illustration, below you can find a brief description of the successful treatment with MTX 15 mg/week in a 27-year-old patient with severe sarcoidosis—massive consolidations in lungs, respiratory failure (MRC 3), and moderate impairment of pulmonary ventilation and diffusion capacity of the lungs. At the same time, the patient had subcompensated diabetes mellitus, which excluded the possibility of CS therapy.

**Figure 1:**
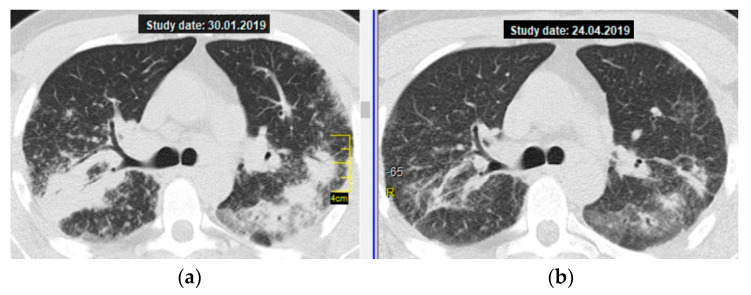
Pulmonary sarcoidosis, stage II, atypical form—multiple massive consolidations in both lungs; a thoracic CT scan (axial section at the level of the tracheal bifurcation): (**a**) on the left—before treatment, (**b**) on the right—regression after 3 months of MTX therapy (15 mg/week).

The patient underwent monotherapy with MTX at a dose of 15 mg/week. As a result, by the second visit (after 3 months of treatment), a pronounced positive dynamic of subjective symptoms was noted—dyspnea occurred only when performing the usual load (when climbing the stairs to the third or fourth floor), the cough disappeared, and the body temperature returned to normal. Figure 1 shows a thoracic CT scan (axial section at the level of the tracheal bifurcation): the almost complete resolution of consolidations in S_3,6_ of both lungs is clearly visible.

The tolerability of MTX was satisfactory: there were no gastrointestinal disturbances; at the seventh month of the treatment period, when conducting a clinical blood analysis, moderate leukocytopenia and mild thrombocytopenia were found, while the value of these parameters did not require dose adjustment and the regimen of MTX use.

**Figure 2:**
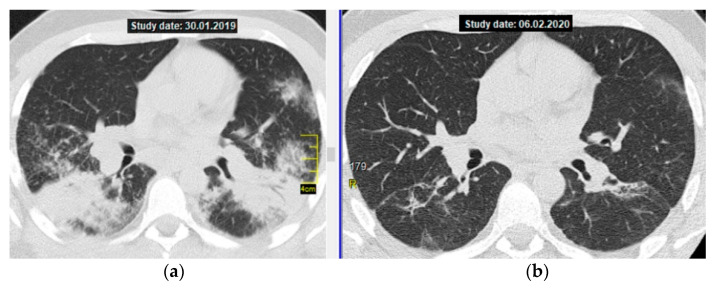
Pulmonary sarcoidosis, stage II, atypical form—multiple massive consolidations in both lungs; a thoracic CT scan (axial section at the level of division of the main bronchi): (**a**) on the left—before treatment, (**b**) on the right—after 12 months of MTX therapy, 15 mg/week (complete resolution with residual changes in the form of limited interstitial fibrosis).

Figure 2 shows CT axial sections, on the left—on the day of diagnosis, on the right—after the end of methotrexate therapy.

Thus, increasing the MTX dose from 10 to 15 mg/week accelerates the rate of regression of sarcoidosis, improves treatment efficacy, and does not affect the rate of serious side effects.

### 3.4. Relapse Rate after Successful Management

A relapse is considered as a recurrence of clinical and/or radiological symptoms after successful management with the normalization of CT findings.

The patients were invited to visit the clinic for assessments using CT 6 and 12 months after the end of treatment. Findings on the relapse rate from 60 post-CS patients and 24 post-MTX patients were collected.

Post-MP relapses were reported in 26 (43.3%) patients, and for post-MTX, in 3 (8.3%; Chi-square = 9.450, *p* = 0.003).

## 4. Discussion

Historically, CS are the first-line drugs in sarcoidosis management due to their sufficiently high efficacy [8,11,12,13,25,26]. Overall, a response to CS is obtained in 80% to 90% of patients, from a few weeks (3 to 4 weeks in case of lung involvement [27]) to 3 months, with a complete response often obtained at 6 weeks [28].

Second-line drugs, with MTX being the most common, are considered if CS treatment is contraindicated, and in the case of steroid-resistance and steroid-induced serious adverse effects.

In 2013, WASOG experts published multinational evidence-based World Association of Sarcoidosis and Other Granulomatous Disorders recommendations for the use of methotrexate in sarcoidosis: integrating systematic literature research and expert opinions of sarcoidologists worldwide [18]; however, only one randomized controlled MTX efficacy study in pulmonary sarcoidosis was conducted at this point [29].

The limitation for a wider MTX prescription in pulmonary sarcoidosis patients is due to a common perception among practicing pulmonologists that MTX, as opposed to CS, results in a less pronounced clinical effect and is associated with serious hepatotoxicity and haematotoxicity. In recent years, rebuttal clinical studies were conducted [22,23,30].

A retrospective methotrexate efficacy and tolerability study in pulmonary sarcoidosis patients conducted by Fang, C. et al. [22] has shown a good clinical response to MTX in 80% of patients. Good drug tolerability and low drug withdrawal rates were observed in these patients even without folic acid supplements in clinical practice.

A large retrospective study by Baughman, R.P. et al. [23] assessed haematotoxicity and hepatotoxicity of methotrexate at a dose of 10 mg weekly in 607 sarcoidosis patients over a 6-year period. Leukopenia and elevated hepatic transaminases were reported in about 10% of cases. Only one patient had severe leukopenia. Only nine patients presented with transaminases elevated > 3 × ULN. Methotrexate was effective in the majority of these patients. The authors did not reveal other adverse effects resulting in methotrexate withdrawal at that time.

The results of a retrospective observational study in 1276 sarcoidosis patients conducted by Vizel’, A.A. et al. [30] allowed them to conclude that 15 mg MTX weekly per os in patients with advanced sarcoidosis who were previously on systemic CS is an effective second-line drug. The sufficient safety level of MTX allows its long-term prescription from 3 months to ≥ 1 year.

Our findings are in line with the above data on methotrexate efficacy and tolerability in the management of patients with pulmonary sarcoidosis. Meanwhile, we have shown that MTX did not significantly differ from treatment using an MP first-line drug in terms of clinical response and the rate of severe adverse effects requiring drug discontinuation. At the same time, cases of resistance to MTX were reported as substantively rare versus CS therapy at the stage of MP dose reduction.

A high relapse rate after CS therapy is one of the main challenges in the management of sarcoidosis patients [14].

In 1997, J.E. Gottlieb et al. published a paper [15] which provides observations from 337 patients with different stages of sarcoidosis over 4 years. The authors have established a significantly higher relapse rate in patients treated with CS (74%) versus those who were not prescribed these drugs (8%). They came to a conclusion that long-term CS treatment may promote increased relapse risk.

It was also found that relapses were more common over the 12 months after the end of treatment [14], as well as in patients on higher CS doses [16,31].

In our study, the relapse rate over the 12 months after a successful MTX treatment was significantly lower versus post-MP patients, which is in line with literature findings on a low relapse rate after MTX treatment [22,23] and confirms a hypothesis, at least partially, that long-term CS therapy is a risk factor for sarcoidosis relapse. In our opinion, this is the most significant result of our study.

### Limitations of the Study

This is a real-life observational study, that is, non-randomized and uncontrolled. Only two main inclusion criteria were used: newly diagnosed pulmonary sarcoidosis without prior specific therapy, and the absence of fibrosis on CT. In addition, in contrast to the CS group, 50% of patients in the MTX group had concomitant diseases or contraindications to CS, which could affect the effectiveness and tolerability of MTX. Furthermore, the limited number of observations in the study of the dependence of outcomes on the dose of MTX did not provide statistically flawless evidence.

## 5. Conclusions

We believe that the literature data provided [22,23,30] and the results of our own MTX and MP efficacy and tolerability studies in patients with pulmonary sarcoidosis may be helpful for practicing pulmonologists in the objective assessment of the risk–benefit ratio in the case of relative contraindications or limitations to the use of CS therapy and they may choose the appropriate treatment agent.

## Figures and Tables

**Table 1:** Baseline clinical, functional, and CT symptoms.

Symptoms	MP (*n* = 97)	MTX (*n* = 46)	Chi-Squared Test
Clinical symptoms:	73 (75.3%)	39 (84.9%)	1.667 *p* = 0.197
Generalized weakness	58 (59.8%)	30 (65.2%)	0.388 *p* = 0.534
Dyspnea on moderate exertion	24 (24.7%)	12 (26.1%)	0.030 *p* = 0.863
Dyspnea on mild exertion	8 (8.25)	5 (10.8%)	0.260 *p* = 0.611
Cough	39 (40.2%)	21 (45.7%)	0.380 *p* = 0.538
Sub-febrile body temperature	17 (17.5%)	6 (13.0%)	0.464 *p* = 0.494
Pulmonary ventilation disturbances	17 (17.5%)	12 (26.1%)	1.415 *p* = 0.235
Decreased DLCO (range, 61–79% predicted)	13 (13.4%)	9 (19.6%)	0.910 *p* = 0.340
CT symptoms:			
Classical findings—bilateral hilar lymphadenopathy and micronodular pattern with perilymphatic distribution	90 (92.8%)	41 (89.1%)	0.542 *p* = 0.462
Uncommon findings—macronodules, consolilations	7 (7.2%)	5 (10.9%)	0.542 *p* = 0.462

**Table 2:** Primary outcomes in patients with pulmonary sarcoidosis after MP and MTX therapy.

Primary Outcomes	MP (*n* = 97)	MTX (*n* = 46)	Chi-Squared Test
Successful completion of treatment	68 (70.1%)	29 (63.0%)	0.703 *p* = 0.399
Lack of efficacy	7 (7.2%)	10 (21.7%)	6.283 *p* = 0.013 *
Progression during treatment	17 (17.5%)	2 (4.3%)	4.703 *p* = 0.031 * Fisher’s exact test: *p* = 0.035 *
Serious side effects	5 (5.2%)	5 (10.9%)	1/567 *p* = 0.211

Note: *—the difference in the value of the indicator in the groups is statistically significant with a critical level of significance = 0.05.

**Table 3:** Primary outcomes of patients with pulmonary sarcoidosis depending on the dose of MTX.

Primary Outcomes	(MTX—10 mg/Week), *n* = 28	(MTX—15 mg/Week), *n* = 18	Chi-Squared Test
Successful completion of treatment	14 (50.0%)	15 (83.3%)	5.225 * p = 0.023
Lack of efficacy	9 (32.1%)	1 (5.6%)	4.552 * p = 0.033
Progression during treatment	1 (3.6%)	1 (5.6%)	0.104 *p* = 0.748
Serious side effects	4 (14.3%)	1 (5.6%)	2.090 *p* = 0.149

Note: *—the difference in the value of the indicator in the groups is statistically significant with a critical level of significance = 0.05.

## Data Availability

For data availability, contact the corresponding author.

## Abbreviations

MTX	methotrexate
MP	methylprednisolone
CT	computed tomography
CS	corticosteroids
DLCO	diffusing capacity of the lung for carbon monoxide
WASOG	World Association of Sarcoidosis and Other Granulomatous Disorders
ALT	alanine aminotransferase
PFTs	pulmonary functional tests
